# Sinopyrophorinae, a new subfamily of Elateridae (Coleoptera, Elateroidea) with the first record of a luminous click beetle in Asia and evidence for multiple origins of bioluminescence in Elateridae

**DOI:** 10.3897/zookeys.864.26689

**Published:** 2019-07-17

**Authors:** Wen-Xuan Bi, Jin-Wu He*, Chang-Chin Chen, Robin Kundrata, Xue-Yan Li

**Affiliations:** 1 State Key Laboratory of Genetic Resources and Evolution, Kunming Institute of Zoology, Chinese Academy of Sciences, Kunming 650223, China; 2 Room 401, No. 2, Lane 155, Lianhua South Road, Shanghai, 201100, China; 3 NPS office, Tianjin New Wei San Industrial Company, Ltd., Tianjing, China; 4 Department of Zoology, Faculty of Science, Palacky University, 17. listopadu 50, 77146, Olomouc, Czech Republic

**Keywords:** China, mitochondrial genome, molecular phylogeny, new genus, new species, taxonomy

## Abstract

The new subfamily Sinopyrophorinae within Elateridae is proposed to accommodate a bioluminescent species, *Sinopyrophorusschimmeli* Bi & Li, **gen. et sp. nov.**, recently discovered in Yunnan, China. This lineage is morphologically distinguished from other click-beetle subfamilies by the strongly protruding frontoclypeal region, which is longitudinally carinate medially, the pretarsal claws without basal setae, the hind wing venation with a well-defined wedge cell, the abdomen with seven (male) or six (female) ventrites, the large luminous organ on the abdominal sternite II, and the male genitalia with median lobe much shorter than parameres, and parameres arcuate, with the inner margin near its apical third dentate. Molecular phylogeny based on the combined 14 mitochondrial and two nuclear genes supports the placement of this taxon far from other luminescent click-beetle groups, which provides additional evidence for the multiple origin of bioluminescence in Elateridae. Illustrations of habitus and main diagnostic features of *S.schimmeli* Bi & Li, **gen. et sp. nov.** are provided, as well as the brief description of its luminescent behavior.

## Introduction

The cosmopolitan family Elateridae currently contains approximately 600 genera and almost 10,000 species (Costa et al. 2010, Kundrata and Bocak 2011). Based on a synthesis of the work over the past several decades, Costa et al. (2010; and references therein) provided the main characteristics of adult and larval elaterid morphology and recognized 17 subfamilies. Thereafter, slight modifications of the above classification were proposed by Kundrata and Bocak (2011), Kundrata et al. (2016, 2018), Bocak et al. (2018), and Douglas et al. (2018) based on molecular analyses. Currently, 18 subfamilies and 37 tribes of Elateridae are recognized (Kundrata et al. 2018).

Approximately 200 species of Elateridae are able to emit light, and these belong to Agrypninae: Pyrophorini (most species), Thylacosterninae (*Balgusschnusei* Heller) and Campyloxeninae (*Campyloxenuspyrothorax* Fairmaire) (Costa et al. 2010). The vast majority of bioluminescent species is known from the Neotropical region, with several species occurring in small Melanesian islands (Costa 1975, 1984b; Costa et al. 2010). The position of the luminous organs varies among adults of different elaterid lineages; they can be found on both prothorax and abdomen (Pyrophorini: Hapsodrilina and Pyrophorina), only on the prothorax (*Balgus* Fleutiaux, *Campyloxenus* Fairmaire, Pyrophorini: Nyctophyxina), or only on the abdomen (Pyrophorini: *Hifo* Candèze) (Costa 1975; 1984a, b; Costa et al. 2010).

In 2017, during an expedition to the western Yunnan in China, in which the first author participated, a remarkable dusk-active bioluminescent click beetle with a single luminous organ on the abdomen was discovered. Since no bioluminescent representative of Elateridae has been recorded in Asia to date, morphological study and molecular phylogenetic analysis were undertaken simultaneously to clarify the identity and phylogenetic placement of the new taxon. He et al. (2019) published the mitochondrial genome of this species and provided a preliminary phylogenetic hypothesis for it based on the analysis of 13 protein-coding genes. Here, we formally describe this species in a new genus, which we propose to place into a new subfamily within Elateridae, and provide more robust phylogenetic hypothesis for this taxon.

## Materials and methods

### Morphology

All specimens of the new species were collected from the vicinity of Longchuan County and Yingjiang County in western Yunnan, in subtropical evergreen broadleaf forests by searching for flashes or by setting light traps during the night. Specimens are deposited in the following collections: Kunming Institute of Zoology, Chinese Academy of Sciences, Kunming, China (**KIZ-CAS**), The Insect Collection of Shanghai Normal University, Shanghai, China (**SNUC**), collection of Wen-Xuan Bi, Shanghai, China (**CBWX**), and the collection of Chang-Chin Chen, Tianjin, China (**CCCC**). Morphological terminology follows Calder (1996) and Costa et al. (2010), except for the wing venation, for which we follow Kukalova-Peck and Lawrence (1993, 2004).

### Phylogenetic analysis

To explore the phylogenetic position of the new Chinese luminescent click beetle, we newly generated 14 mitochondrial genes (13 protein-coding genes and *16S*) and two nuclear rRNA genes (*18S* and *28S*) for this species and merge them with the available four-gene Elateridae dataset (*18S*, *28S*, *16S*, *cox1*) by Kundrata et al. (2016, 2018) and Douglas et al. (2018) plus all publicly available Elateridae mitochondrial genomes downloaded from the GenBank (altogether 179 terminals representing 13 subfamilies). Four species of Phengodidae were used as outgroups following Kundrata et al. (2016, 2018) (Suppl. material S1, File 1).

The mitogenome of the new species was obtained from GenBank (accession number MH065615) (He et al. 2019). Because we were not able to obtain high quality reference sequences of *18S* and *28S* from the Illumina reads previously used for assembling the mitogenome, we assembled *18S* and *28S* based on another batch of Illumina reads sequenced using fresh specimens collected from type locality later in June 13, 2018, and immediately frozen in liquid nitrogen and stored at -80 °C before use. Total genomic DNA (gDNA) was isolated from two male adults with Sodium Dodecyl Sulfonate method. Library (150-bp insert size) was prepared and sequenced on the Illumina HisSeq4000. Total 38 Gb clean reads were used for assembling *18S* and *28S* based on the following method: 1) reads were mapped to reference genes of rDNA using mrsFAST v3.3.0 with several iterations (Hach et al. 2014) (first iteration referred to genes downloaded from GenBank (NCBI Resource Coordinators 2016); later iterations referred to assembled contigs in the latest iteration); 2) mapped reads were then assembled using SPAdes v3.11 (Bankevich et al. 2012); 3) tandem contigs were filtered using mereps v2.6 because a huge number of repetitive reads greatly lowers assembly efficiency (Kolpakov et al. 2003); 4) steps one to three were automatically performed for 3–10 iterations until all genes were recovered.

The individual genes were aligned using Mafft online version 7 (https://mafft.cbrc.jp/alignment/server/) (Kuraku et al. 2013) with default parameters. The alignment was displayed and manually curated in Mega7 (Kumar et al. 2016). All 16 aligned genes were concatenated using SequenceMatrix version 1.7.8 (Vaidya et al. 2011). The concatenated matrix was used to calculate the best-fit evolutionary model (GTR+I+G) using PartitionFinder 2.1.1 (Lanfear et al. 2012, 2017). Maximum likelihood (ML) analysis were carried out using RAxML version 7.0.4. (Stamatakis et al. 2008) with 1000 bootstrap replicates. Phylograms were drawn using Interactive Tree of Life (ITOL) (Letunic and Bork 2016).

## Results

### Phylogenetic inference

**Figure 1.**
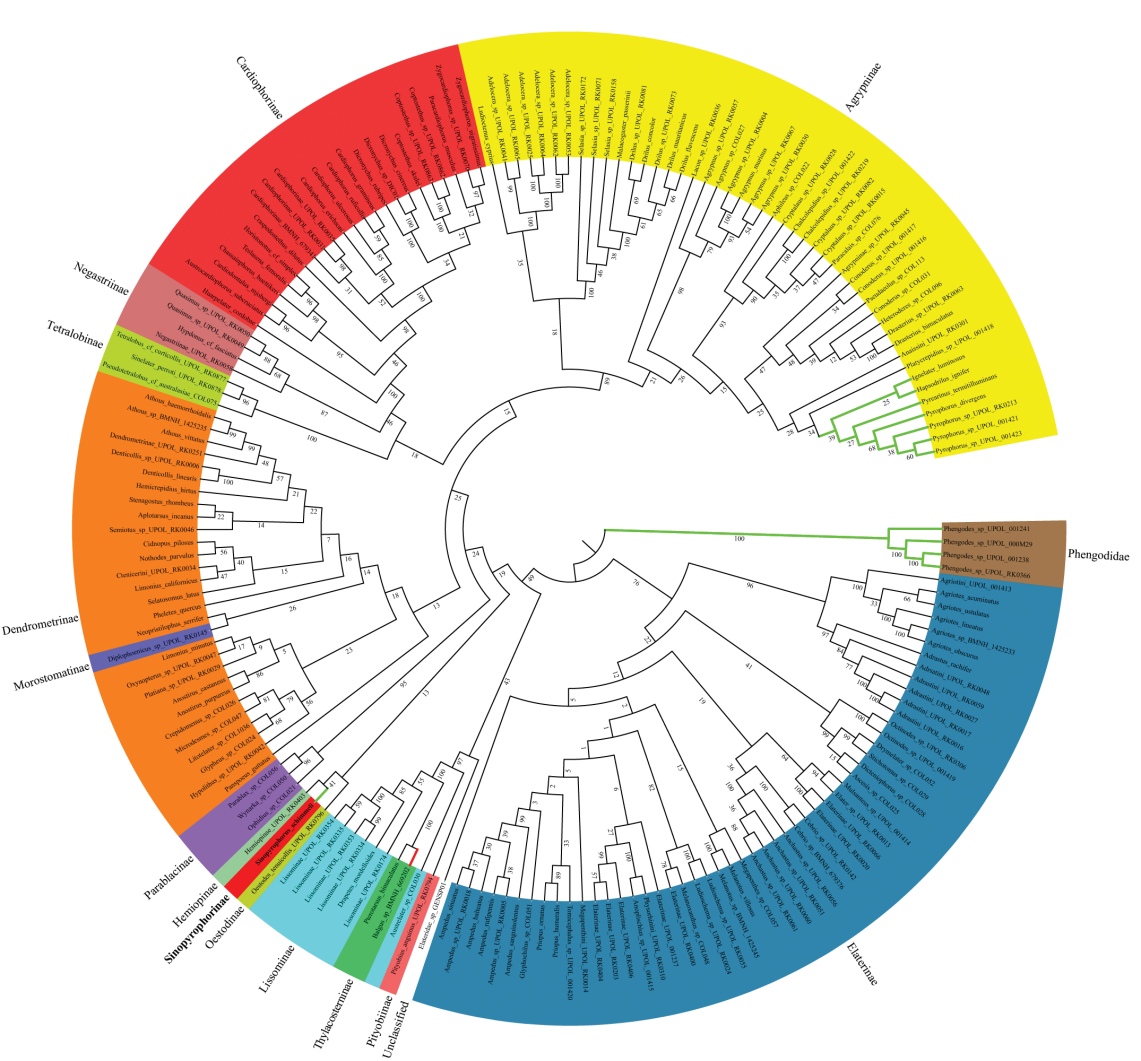
Inferred phylogenetic position of *Sinopyrophorusschimmeli* Bi & Li, gen. et sp. nov. within Elateridae based on the concatenated 14 mitochondrial genes (13 protein-coding genes and *16S*) and two nuclear ribosomal genes (*18S*, *28S*) using the Maximum Likelihood (ML) analysis. Numbers near each branch indicate ML bootstrap values with 1000 replicates. The same colored shaded areas at the terminals denote the same subfamily. Green bold lines indicate luminescent taxa. The bold red line indicates the presence of luminescent species within the same genus.

Our phylogenetic analysis recovered similar topology (Fig. 1) to that of Kundrata et al. (2018), only Tetralobinae were placed in an unsupported terminal clade with Cardiophorinae + Negastriinae, and Dendrometrinae including *Diplophoenicus* Candèze (Morostomatinae) formed a separate clade. Similar to previously published phylogenies, we obtained low statistical support for the backbone of tree. All subfamilies were monophyletic, except for Dendrometrinae (due to the inclusion of *Diplophoenicus*) and Lissominae (due to the inclusion of Thylacosterninae). The newly sequenced luminescent species from China was found in an unsupported clade with Oestodinae and Hemiopinae as follows: *Oestodestenuicollis* (Randall) + (*Hemiops* sp. + new luminescent taxon), far from other bioluminescent groups like Thylacosterninae and Agrypninae: Pyrophorini.

### Taxonomy

#### Sinopyrophorus

Taxon classificationAnimaliaColeopteraElateridae

Bi & Li gen. nov.

45769610-69ac-46cf-be7b-c7889e685617

http://zoobank.org/6828CC35-47EA-4F3A-994E-4BE4C2C4AF8A

Figs 2–3 , 4–16 , 17–23

= SinopyrophorusHe et al., 2019: 565 [nomen nudum; published without description, unavailable name according to the ICZN (1999, Art. 13)].

##### Type species.

*Sinopyrophorusschimmeli* Bi & Li, sp. nov., here designated.

##### Diagnosis.

Head with frontoclypeal region (Fig. 4) strongly protruding, longitudinally strongly carinate medially; antennomeres II and III short, subequal in length; clicking mechanism (i.e., prosternal process fitting into mesoventral cavity) fully developed; prosternal process straight in lateral view, pretarsal claw (Fig. 13) lacking setae at base; hind wing (Fig. 14) with well-defined wedge cell; abdomen with seven (male) or six (female) ventrites; large transverse luminous organ present on abdominal sternite II (Fig. 16); aedeagus (Fig. 20) with parameres arcuate and median lobe much shorter than parameres.

**Figures 2–3.**
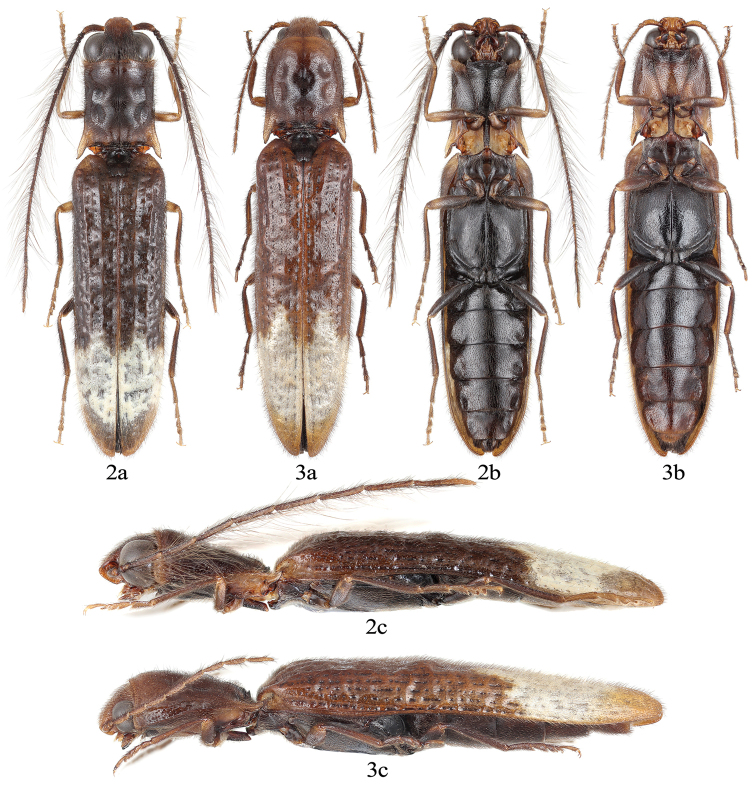
Habitus of *Sinopyrophorusschimmeli* Bi & Li, gen. et sp. nov. paratypes **2** male **3** female. **a**, dorsal view; **b**, ventral view; **c**, lateral view.

**Figures 4–16.**
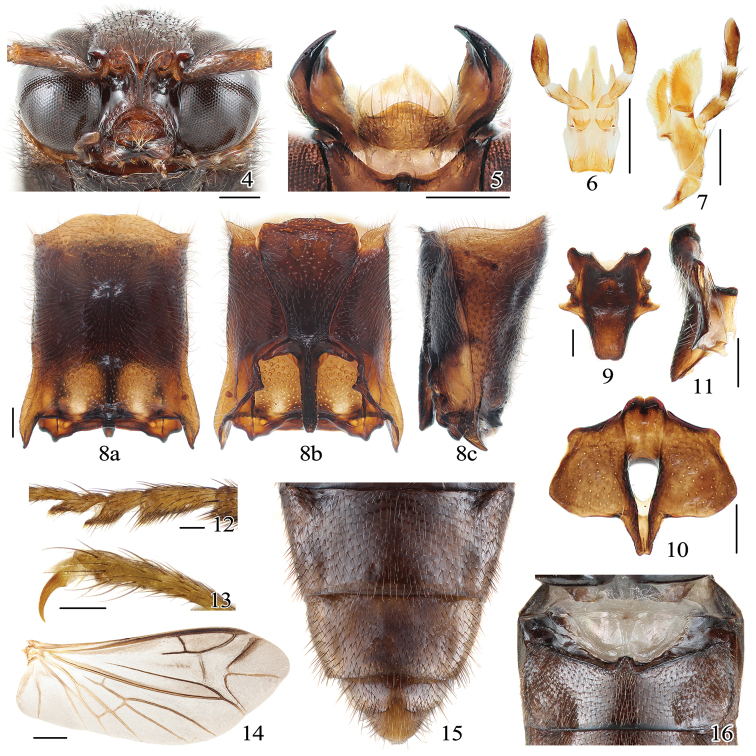
*Sinopyrophorusschimmeli* Bi & Li, gen. et sp. nov. Male **4** head (anterior view) **5** labrum and mandibles (dorsal view) **6** labium **7** maxilla **8** prothorax **9** scutellum (dorsal view) **10** mesoventrite (ventral view) **11** mesoventrite (lateral view) **12** tarsomeres II–IV (lateral view) **13** tarsal claw (lateral view) **14** hind wing **15** ventrites IV–VII **16** abdominal luminescent organ (pale area above ventrite I). **a**, dorsal view; **b**, ventral view; **c**, lateral view. Scale bars: 0.25 mm (**4–11**); 0.1 mm (**12, 13**); 1 mm (**14**); not to scale (**15, 16**).

**Figures 17–23.**
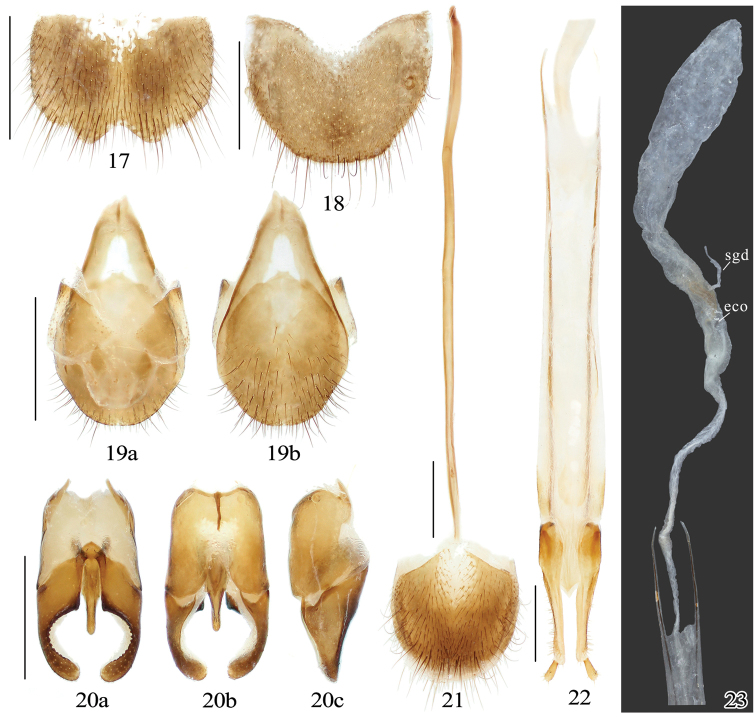
*Sinopyrophorusschimmeli* Bi & Li, gen. et sp. nov. Male **17** sternite VIII **18** tergite VIII **19** tergites IX–X with sternite IX **20** aedeagus. Female **21** sternite VIII **22** ovipositor (dorsal view) **23** internal genital tract. Abbreviations: eco, the entry of the common ovdiduct; sgd, spermathecal gland duct. a, dorsal view; b, ventral view; c, lateral view. Scale bars: 1 mm; not to scale (**23**).

##### Description.

***Male.*** Body elongate, ~ 4.6 times as long as wide, weakly convex in lateral view. Vestiture of fine, suberect setae.

Head with frontoclypeal region strongly protruding, inflexed at apex, medially longitudinally carinate; carina setose and apparently not joined to supra-antennal carinae, basally half as wide as frons, then narrowed and subparallel-sided, with cuticle between edges flat (Fig. 4). Antennal insertions concealed from above. Labrum free, transverse, anterior margin convex in dorsal view. Maxilla (Fig. 7) with galea scoop-like, anterior part covered with setae, denser on inner edge; lacinia elongate, densely pilose; palp with apical palpomere slightly expanded anteriorly. Labium (Fig. 6) with prementum elongate, trilobed anteriorly. Mandibles (Fig. 5) bidentate, apical tooth narrowly acute, subapical mesal tooth small. Antenna with 11 antennomeres, filiform; antennomeres II and III subequal in length (together 1/3 as long as antennomere IV), globular; remaining antennomeres at least five times longer than wide.

Prothorax with chin piece of prosternum short, bisinuate, not concealing labium; prosternal process slightly constricted between coxae in ventral view, almost straight in lateral view. Pronotosternal suture almost straight. Procoxae narrowly separated, externally broadly open. Scutellar shield (Fig. 9) trapezoidal, moderately elevated, narrowed posteriorly, posterior apex slightly emarginate. Mesocoxal cavities narrowly separated, open laterally to both mesepimeron and mesepisternum; mesotrochantin visible. Mesoventrite (Figs 10, 11) with posterior area lower than metaventrite. Meso-metaventral suture distinct. Metacoxae extending laterally to meet metepimeron, metacoxal plates not covering trochanters when legs withdrawn. Hind wing (Fig. 14) 2.35 times as long as wide; apical field ~ 0.25 times as long as total wing length, apical field with three sclerites forming an epsilon figure; radial cell longer than wide, with inner posterobasal angle acute; cross-vein r3 horizontal; MP3+4 with basal cross-vein and basal spur; CuA2 meeting MP4; wedge cell present, ~ 3.5 times as long as wide, with obliquely truncate apex. Leg with trochanter-femur joint oblique; tibial spurs double, tarsal formula 5-5-5; pretarsal claws (Fig. 13) simple, lacking setae at base; empodium weakly developed, bisetose.

Abdomen with seven ventrites (sternites III–IX, Figs 2b, 15). Sternite II with transverse, semicircular luminescent organ occupying more than half of its width (Fig. 16). Intercoxal process of ventrite I (i.e., sternite III) narrowly rounded. First five ventrites subequal in length; ventrite V with posterior margin emarginate. Tergite VIII (Fig. 18); sternite VIII (Fig. 17) ~ 0.7 times as long as ventrite V (i.e., sternite VII), posteromedially emarginate, with each lobe slightly emarginate posteriorly; sternite IX (Fig. 19b) acute at base, more sclerotized at anterior half, connected to tergite IX by membrane; tergites IX and X partly fused (Fig. 19a), narrowly pointed anteriorly. Aedeagus (Fig. 20) with median lobe very short, only approximately half as long as aedeagus, with short basal struts; basal half wider, apical half narrow, slightly concave to subparallel-sided, with rounded apex.

***Female.*** Slightly larger than male. Abdomen with six ventrites. Sternite VIII (Fig. 21) with spiculum ventrale 0.8 times total length of sternite. Ovipositor (Fig. 22) long, with paraprocts 3.5 times longer than gonocoxites; gonocoxites partially sclerotized; styli attached subapically. Internal genital tract (Fig. 23) simple; vagina long, membranous, slightly enlarged near entry of common oviduct; bursa copulatrix elongate, slightly widened anteriorly, sclerotized near base, with single spermathecal gland duct and fine spinules internally; colleterial gland absent.

##### Etymology.

The generic name is derived from the Latin prefix *sino*-, which means Chinese, and *Pyrophorus*, a bioluminescent click-beetle genus from Central and South America. Gender masculine.

##### Distribution.

China: Western Yunnan.

#### Sinopyrophorus schimmeli

Taxon classificationAnimaliaColeopteraElateridae

Bi & Li sp. nov.

36982589-1a66-4036-bc54-250cf14d28a3

http://zoobank.org/D8B48DF6-3B0D-45E4-94DD-127CC4C48B43

Figs 2–3 , 4–16 , 17–23

= SinopyrophorusschimmeliHe et al., 2019: 565 [nomen nudum; published without description, unavailable name according to the ICZN (1999, Art. 13)].

##### Type locality.

China, Yunnan, Yingjiang, Shangbangzhong, 24°26'N, 97°45'W, 1650 m.

##### Type material.

Holotype: male, “China, Yunnan, Yingjiang, Shangbangzhong, 24°26’N, 97°45’W, 1650 m, 2017.VI.23, leg. Wen-Xuan Bi”; “Holotype *Sinopyrophorusschimmeli* sp. nov.” [red handwritten label] (SNUC). Paratypes (9 males, 3 females): 1 female, same data as holotype (KIZ-CAS); 1 male, 1 female, “China, Yunnan, Longchuan, Husa, 1770 m, 2017.VI.13–14, leg. Wen-Xuan Bi” (CBWX); 1 male, ditto except 2000 m, 2017.VI.16 (CBWX); 1 male, 1 female, ditto except 1700 m, 2017.VI.24, leg. Yu-Tang Wang (CBWX); 2 males, ditto except 1770 m, 2016.V.31 (CCCC); 1 male, ditto except leg. Xiao-Dong Yang (CCCC); 3 males, ditto except 2017.VI.25, leg. Wen-Xuan Bi (KIZ-CAS).

##### Other material examined.

2 males, China, Yunnan, Longchuan, Husa, 1770 m, 2017.VI.13–16, leg. Wen-Xuan Bi, damaged, partially used for extracting genomic DNA in a project of mitogenome (accession number MH065615; He et al. 2019); 2 males, China, Yunnan, Longchuan, Husa, 1770 m, 2018.VI.13, leg. Wen-Xuan Bi, the whole body of both specimens was used for the extraction of genomic DNA in an ongoing project of de novo genome sequencing and assembly of 18S and 28S.

##### Diagnostic description.

***Male*** (Fig. 2). Body length 9.6–11.3 mm (holotype: 11.3 mm). Body brown to dark brown, with posterior portion of prothorax, fore- and mid-legs paler; elytra with broad subapical pale band, zigzagged anteriorly, rounded posteriorly. Body surface with fine, suberect brown setae, denser on legs; elytral light band with pale setae.

Head transverse, weakly convex, 0.75 times as long as wide, same width as pronotal anterior edge; sparsely and finely punctate. Frons rectangular, 1.4 times longer than width, 0.3 times as wide as head width across the eyes. Frontoclypeal region concave at sides beneath; with one small median depression. Eyes protuberant, median width of each eye ~ 0.7 times interocular distance in dorsal view. Mouthparts directed anteroventrally. Labrum (Fig. 5) 2.2 times wider than long. Antenna long, reaching second half of elytral length, ~ 0.7 times as long as body length; scape 2.3 times as long as combined length of antennomeres II and III, and of approximately same length as antennomere 4, slightly curved; antennomeres 4–11 successively weakly lengthened, with fine and very long, distinct setae; setae almost as long as apical antennomere.

Prothorax (Fig. 8) slightly convex in lateral view, tallest anteriorly; ~ 1.2 times as long as wide in dorsal view, weakly narrowed anteriorly, slightly narrower than elytral humeral width; sides slightly sinuate; pronotal lateral carina complete; anterior angles short, subacute; hind angles narrowly acute, moderately produced posterolaterally, each with short carina; posterior edge straight from dorsal view, medially elevated; pronotal disk sparsely and finely punctate, with eight shallow depressions: single median and posteromedian, and three pairs at sides. Prosternum and hypomeron more coarsely punctate than pronotum. Elytra ~ 3.0 times as long as combined width, ~ 2.9 times as long as pronotum, parallel-sided; each elytron with three low and evenly spaced swellings, longitudinally arranged at basal half near suture; with nine punctate striae; apices conjointly rounded; epipleura short, abruptly narrowed near metacoxa. Legs long; tarsomeres I–III elongate, tarsomere I ~ 1.4 times as long as tarsomere II, tarsomere II as long as combined lengths of tarsomeres III and IV or as long as tarsomere V; tarsomeres III and IV ventrally lobate (Fig. 12).

Abdomen with each ventrite with paired depressions posterolaterally. Aedeagus (Fig. 20) robust, ~ 1.6 times as long as wide. Phallobase slightly longer than wide, narrowed dorsally, emarginate posteriorly. Median lobe approximately half as long as aedeagus, with basal struts 0.25 times total length of median lobe. Parameres ~ 1.5 times longer than median lobe, shorter than phallobase, with dorsal surface ~ 1.6 times longer than ventral one, partially fused basally in dorsal view; each paramere with dorsal surface angulate on inner margins at basal 2/5, strongly narrowed and curved mesad with dentate inner margin near apical 1/3 and rounded apex; bearing 20–30 fine setae near apex, longer on ventral surface.

***Female*** (Fig. 3). Body length 12.1–14.5 mm. Similar to male in its general appearance but with integument paler. Eyes smaller, median width of each eye ~ 0.4 times interocular distance in dorsal view. Antenna shorter, only reaching elytral humeri, ~ 0.3 times as long as body length. Pronotum relatively shorter with lateral margins more rounded, narrowed anteriorly, with rounded anterior angles. Elytra relatively longer, ~ 3.3 times as long as wide, ~ 3.0 times as long as pronotum. Legs relatively shorter. Abdominal luminescent organ smaller, occupying approximately one third of basal abdominal sternite width.

##### Immature stages.

Unknown.

##### Etymology.

This species is named in honor of late Mr. Rainer Schimmel, a specialist in Elateridae, who kindly provided valuable comments at the beginning of this study.

##### Biological notes.

All specimens of the new species were collected during the late May to June (i.e., the middle of the rainy season) from the mountain area in vicinity of Longchuan County or Yingjiang County, western Yunnan in subtropical evergreen broadleaf forests by searching for flashes or by light trapping during night. The adults of both sexes emitted a continuous yellowish green light from the abdominal luminous organs while in flight, or during a short time when preparing for flight or afterwards. During this process the luminous organ is exposed ventrally by raising and extending the abdomen from the metaventrite (Supplementary material S2, file 2). The reaction of the adults when disturbed during a flight is to retract their abdomen, hide their luminous organ, and show a death-feigning behavior (thanatosis). Thanatoid adults remained inactive for a long time, which was obviously different from the observation in Pyrophorini because the latter begin to emit light and are very active after being disturbed (Costa 1975). At least three lampyrid species (*Luciola* sp., *Diaphanes* sp., and *Pyrocoelia* sp.) occurred sympatrically with the new elaterid species and can be found simultaneously during night but can be easily distinguished by different flash patterns. Although some specimens of *S.schimmeli* were previously collected using light traps in 2016, this species was found to be luminescent only in 2017 when its bioluminescent behavior was observed in the field. This was caused by the abdominal luminescent organ being hidden when the beetle is not active.

##### Nomenclatural notes.

He et al. (2019) reported the mitochondrial genome of *S.schimmeli* gen. et sp. nov. and used the genus and species name of the here described taxon in their study. The paper of He et al. (2019) was intended to be published after the formal description of *S.schimmeli* Bi & Li, gen. et sp. nov. but unfortunately, it was published earlier, causing nomenclatural problems by reporting the genus and species names without available descriptions and thus unavailable according to the Code (ICZN 1999).

#### Sinopyrophorinae

Taxon classificationAnimaliaColeopteraElateridae

Bi & Li subfam. nov.

d3630283-caee-40c9-8d7a-94cba0f5f230

http://zoobank.org/8964AB43-FA98-4FB3-8D9A-2E95150E94AE

##### Type genus.

*Sinopyrophorus* Bi & Li, gen. nov., here designated.

##### Diagnosis.

The molecular phylogenetic analysis (Fig. 1) and morphology (Figs 2–23) justify the establishment of a new monogeneric subfamily Sinopyrophorinae Bi & Li, subfam. nov. within Elateridae. Sinopyrophorinae are easily recognizable by the strongly protruding frontoclypeal region (Fig. 4), which is medially distinctly longitudinally carinate, antennomeres II and III subequal in length and together less than half as long as antennomeres IV–XI, pronotal hind angles (Fig. 8) acute, produced posterolaterally, prosternal process (Fig. 8c) straight in lateral view, tarsomeres III and IV (Fig. 12) with ventral lobes, abdomen with seven (male) or six (female) ventrites, with a luminous organ (Fig. 16) on sternite II, and aedeagus (Fig. 20) with a median lobe shorter than phallobase, and arcuate parameres.

## Discussion

### Phylogenetic placement and morphology of *Sinopyrophorusschimmeli* Bi & Li, gen. et sp. nov.

He et al. (2019) provided the first preliminary phylogenetic hypothesis for *S.schimmeli* based on the analysis of 13 protein-coding mitochondrial genes. They found this species sister to a clade containing Dendrometrinae and Elaterinae; however, their sampling of the click-beetle lineages was limited and the position of *S.schimmeli* was not statistically supported.

Here, we used the most comprehensive dataset of Elateridae to date to elucidate the phylogenetic position of the first known Asian luminescent species. Our analysis (Fig. 1) placed *S.schimmeli* in a clade with *Hemiops* and *Oestodes*, which are both the type genera of the subfamilies Hemiopinae and Oestodinae, respectively, and which were not included in the study by He et al. (2019). However, the relationships among these lineages are not statistically supported and the long branches (see Kundrata et al. 2016) indicate a long-term independent evolution of these groups, which is supported by their distinct morphology. Sinopyrophorinae differ from both Hemiopinae and Oestodinae by having the strongly protruding frontoclypeal region with median longitudinal carina, the antennomeres II and III subequal in length and much shorter than remaining antennomeres (present only in the hemiopine genus *Plectrosternus* Lacordaire), the posterior margin of pronotum simple, without sublateral incisions or carinae, the prosternal process straight in lateral view, the posterior end of scutellar shield emarginate, the wide mesoventrite with an elongate mesoventral cavity which is only gradually narrowed posteriorly, the bisetose empodium (present in *Oestodes*, multisetose in Hemiopinae), the abdomen with six or seven ventrites and with a luminous organ, the male genitalia with a median lobe shorter than parameres, and each paramere strongly curved mesad with a dentate inner margin, the female genitalia with a more sclerotized ovipositor, longer paraprocts and styli attached subapically to gonocoxites (apically in *Oestodes* and Hemiopinae; A. Prosvirov, pers. comm.). These morphological differences in combination with the molecular phylogeny support the subfamilial rank of Sinopyrophorinae, especially when there are no obvious synapomorphies which would define the clade of Sinopyrophorinae + Oestodinae + Hemiopinae, or placement of *Sinopyrophorus* in any other subfamily. Since the backbone of the Elateridae phylogenetic tree is not or only weakly supported (Kundrata et al. 2016, 2018; this study), future genomic or transcriptomic data might help to resolve the relationships among the deep splits including Sinopyrophorinae.

Our molecular analysis in combination with the morphological investigation confirmed that *S.schimmeli* does not belong to any of described subfamilies containing bioluminescent species. The clade of Sinopyrophorinae, Oestodinae and Hemiopinae formed one of the basal radiations in Elateridae, far from the Agrypninae: Pyrophorini, which contains the majority of luminescent click beetles (Fig. 1). The position of *S.schimmeli* far from Pyrophorini is additionally supported by the morphological features such as the pretarsal claws without setae at base (present in Pyrophorini), hind wings with a well-defined wedge cell (absent in Pyrophorini), abdomen with six or seven ventrites (five in Pyrophorini) and the presence of styli on the gonocoxites of ovipositor (styli absent in Pyrophorini). Thylacosterninae, which include the luminescent *Balgusschnusei*, are regularly recovered inside Lissominae in recent DNA-based analyses (Kundrata et al. 2014, 2016, 2018; Bocak et al. 2018; this study). They differ from Sinopyrophorinae in having the flabellate antennae, deep antennal cavities lying beneath the hypomera, and membranous tarsal lobes (Costa 1984a, Costa et al. 2010). The members of the subfamily Campyloxeninae, which have not been included in any DNA-based study to date, differ from Sinopyrophorinae by much wider frontoclypeal region with a complete frontal carina (frontoclypeal region strongly protruding, relatively narrow and high, longitudinally carinate medially in *S.schimmeli*), narrowed apical portions of lateral lobes of mesoventrite (wide in *S.schimmeli*) oval-shaped mesoventral cavity (elongate, gradually narrowed posteriorly in *S.schimmeli*), abdomen with five ventrites and without a luminous organ (six or seven ventrites, and a large abdominal luminous organ in *S.schimmeli*), distinctly longer paraprocts, differently shaped aedeagus with the median lobe longer than parameres, and the paramere elongate, with a subapical hook, and apex oriented posteriorly (Costa 1975, Arias-Bohart 2015).

Southern China including Yunnan is a world biodiversity hotspot (Myers et al. 2000) and hosts many endemic beetle species including Elateridae. Intensive entomological research in southern China and neighboring regions is necessary to find out if the newly established Sinopyrophorinae contains more species. Additionally, the discovery of a larva of *S.schimmeli* may help to better understand the morphological distinctiveness and systematic position of this interesting beetle lineage.

### Evolution of bioluminescence in Elateridae

Bioluminescence has evolved independently many times in various organisms (Day et al. 2004). Within Coleoptera, bioluminescence is best known in Lampyridae (fireflies), but also present in Phengodidae, Rhagophthalmidae, and some Elateridae, all within the superfamily Elateroidea (e.g., Oba 2009). Additionally, it was reported also for the larvae of two species belonging to Staphylinidae (Costa et al. 1986, Rosa 2010), which are not closely related to Elateroidea (e.g., Zhang et al. 2018). Within the Elateroidea, bioluminescence independently evolved several times (Bocakova et al. 2007, Sagegami-Oba et al. 2007, Amaral et al. 2014, Fallon et al. 2018) but despite the recent progress in elucidating the phylogenetic relationships within this superfamily (Kundrata et al. 2014), the relationships between the luminescent lineages remain unresolved.

Within Elateridae, nearly all luminescent taxa belong to the agrypnine tribe Pyrophorini, and each of the remaining three smaller groups contain only a single bioluminescent species. All other bioluminescent subfamilies, except Sinopyrophorinae, also include non-luminescent species. Campyloxeninae were placed into Pyrophorini by Stibick (1979) but this was not accepted by later authors (see e.g., Costa et al. 2010). In fact, the members of Campyloxeninae differ diagnostically from Pyrophorini in e.g., the absent setae on tarsal claws, presence of the wedge cell in the hind wing venation and the ovispositor with styli (Costa 1975, Arias-Bohart 2015). Current molecular phylogeny suggests at least three independent origins of bioluminescence within Elateridae, i.e., in Agrypninae: Pyrophorini, Sinopyrophorinae and Thylacosterninae. The presence of a single luminous organ located on abdomen without any prothoracic bioluminescent organs, which is shared by *S.schimmeli* and the pyrophorine genus *Hifo*, is therefore an apparent homoplasy. Sequences of the fresh DNA-grade material of Campyloxeninae would help us to better understand the position of this group and if it represents a fourth origin of bioluminescence in Elateridae. All available phylogenetic analyses indicate that the ancestral state of Elateridae was nonluminescent and the luminescence was later obtained in several independent lineages (Bocakova et al. 2007, Sagegami-Oba et al. 2007, Douglas 2011, Kundrata and Bocak 2011, Kundrata et al. 2014).

In Lampyridae, bioluminescence was first gained by larvae as an aposematic warning display, and subsequently gained by adults and co-opted as a sexual signal; the overall trend of courtship is the use of pheromones in ancestral species, then pheromones used in conjunction with photic signals, then the sole use of photic signal (Branham and Wenzel 2001, 2003; Ohba 2004; Stanger-Hall et al. 2018). Males of *S.schimmeli* have a large luminous organ on abdomen plus very long antennae (longer than in females, hypothesized to be for pheromone detection) and large eyes (larger than in females, and hypothesized to be for bioluminescence detection), which suggest the use of multiple communication channels for mate attraction (Stanger-Hall et al. 2018, and references therein). It is hypothesized that in elaterids, bioluminescence of the abdominal lantern is an optical signal for the intraspecific sexual communication, while the signals from the prothoracic lanterns (if present) serve to warn predators and may also provide illumination in flight (Lall et al. 2000, 2010). Additionally, antennae are usually relatively longer in species which rely greatly on pheromones as sexual signals (Stanger-Hall et al. 2018). Therefore, the external morphology of *S.schimmeli* suggests the combined sexual communication of both light signals and pheromones. The relatively larger sexual signal sensors of *S.schimmeli* males in comparison to conspecific females are in agreement with situation in many other insect groups, in which males are the more actively searching sex, with more sensitive sensors (Steinbrecht 1987, Stanger-Hall et al. 2018).

The discovery of *S.schimmeli* as the first record of a bioluminescent click beetle in Asia shed new light on the geographic distribution and evolution of luminescent click beetles. As a representative of a unique lineage, only distantly related to all other luminescent click beetles, *S.schimmeli* may serve as a new model taxon in the research of bioluminescence within Coleoptera. A project of *de novo* genome sequencing of *S.schimmeli* has already started and should help answer the questions related to the genome characteristics of this taxon (e.g., genome size, heterozygosity etc.), its genomic difference from those of luminescent pyrophorine click beetles, and the genomic basis of the origin of its bioluminescence.

## Supplementary Material

XML Treatment for Sinopyrophorus

XML Treatment for Sinopyrophorus schimmeli

XML Treatment for Sinopyrophorinae

## Supplementary materials

Supplementary material 1 Table 1. Data type: molecular data Explanation note: The GenBank accession numbers of mitochondrial genes (PCGs and *16S*) and nuclear rRNA genes (*18S* and *28S*) in Elateridae and outgroup used for the phylogenetic analysis and references in rRNA assembly. https://binary.pensoft.net/file/318555This dataset is made available under the Open Database License (http://opendatacommons.org/licenses/odbl/1.0/). The Open Database License (ODbL) is a license agreement intended to allow users to freely share, modify, and use this Dataset while maintaining this same freedom for others, provided that the original source and author(s) are credited.Jin-Wu He

Supplementary material 2 Movie 1. Data type: multimedia Explanation note: Luminescent behavior of *Sinopyrophorusschimmeli*. https://binary.pensoft.net/file/318556This dataset is made available under the Open Database License (http://opendatacommons.org/licenses/odbl/1.0/). The Open Database License (ODbL) is a license agreement intended to allow users to freely share, modify, and use this Dataset while maintaining this same freedom for others, provided that the original source and author(s) are credited.Wen-Xuan Bi

Supplementary material 3 Sequence/matrix data. Data type: multimedia Explanation note: Luminescent behavior of *Sinopyrophorusschimmeli*. https://binary.pensoft.net/file/318557This dataset is made available under the Open Database License (http://opendatacommons.org/licenses/odbl/1.0/). The Open Database License (ODbL) is a license agreement intended to allow users to freely share, modify, and use this Dataset while maintaining this same freedom for others, provided that the original source and author(s) are credited.Wen-Xuan Bi
